# Identification of Differentially Expressed Non-coding RNA in Porcine Alveolar Macrophages from Tongcheng and Large White Pigs Responded to PRRSV

**DOI:** 10.1038/s41598-018-33891-0

**Published:** 2018-10-23

**Authors:** Yueran Zhen, Fengqing Wang, Wan Liang, Jianjian Liu, Guoli Gao, Yan Wang, Xuewen Xu, Qiuju Su, Qingde Zhang, Bang Liu

**Affiliations:** 1 Key Laboratory of Agricultural Animal Genetics, Breeding and Reproduction of Ministry of Education, Key Laboratory of Pig Genetics and Breeding of Ministry of Agriculture & College of Animal Science and Technology, Huazhong Agricultural University, Wuhan, 430070 China; 2 The Cooperative Innovation Center for Sustainable Pig Production, Wuhan, 430070 China; 3 Laboratory Animal Center, College of Animal Science and Technology & Veterinary Medicine, Huazhong Agricultural University, Wuhan, 430070 China

## Abstract

Porcine reproductive and respiratory syndrome (PRRS) is one of the most ruinous diseases in pig production. Our previous work showed that Tongcheng pigs (TC) were less susceptible to PRRS virus (PRRSV) than Large White (LW) pigs. To elucidate the difference in PRRSV resistance between the two breeds, small RNA-seq and ribo-zero RNA-seq were used to identify differentially expressed non-coding RNAs (including miRNAs and lincRNAs) responded to PRRSV in porcine alveolar macrophages (PAMs) from TC and LW pigs. Totally, 250 known mature miRNAs were detected. For LW pigs, there were 44 down-regulated and 67 up-regulated miRNAs in infection group; while for TC pigs, 12 down-regulated and 23 up-regulated miRNAs in TC infection group were identified. The target genes of the common differentially expressed miRNAs (DEmiRNAs) in these two breeds were enriched in immune-related processes, including apoptosis process, inflammatory response, T cell receptor signaling pathway and so on. In addition, 5 shared DEmiRNAs (miR-181, miR-1343, miR-296-3p, miR-199a-3p and miR-34c) were predicted to target PRRSV receptors, of which miR-199a-3p was validated to inhibit the expression of CD151. Interestingly, miR-378 and miR-10a-5p, which could inhibit PRRSV replication, displayed higher expression level in TC control group than that in LW control group. Contrarily, miR-145-5p and miR-328, which were specifically down-regulated in LW pigs, could target inhibitory immunoreceptors and may involve in immunosuppression caused by PRRSV. This indicates that DEmiRNAs are involved in the regulation of the immunosuppression and immune escape of the two breeds. Furthermore, we identified 616 lincRNA transcripts, of which 48 and 30 lincRNAs were differentially expressed in LW and TC pigs, respectively. LincRNA *TCONS_00125566* may play an important role in the entire regulatory network, and was predicted to regulate the expression of immune-related genes through binding with miR-1343 competitively. In conclusion, this study provides an important resource for further revealing the interaction between host and virus, which will specify a new direction for anti-PRRSV research.

## Introduction

Porcine reproductive and respiratory syndrome (PRRS), caused by PRRS virus (PRRSV), is an immunosuppressive disease characterized by reproductive disorder of breeding sows and respiratory symptoms of piglets. It has always been a major threat to pig industry since its first outbreak in the 1980s. The infection of PRRSV is mediated by specific receptors, including heparan sulfate, sialoadhesin, CD163, vimentin, CD151, and CD209^1–6^. Porcine alveolar macrophages (PAMs) are the primary target cells for PRRSV to replicate *in vivo*^7^. As a single-strand RNA virus, PRRSV genome mutates with a high rate. Due to the poor cross-protection of the traditional vaccine for PRRSV variants, clinical prevention of PRRS is quite difficult, thus host genetic improvement of PRRSV resistance would be a better choice. As early in 1998, Harbul *et al*. reported that genetic differences of host could affect the susceptibility to PRRSV and clinical symptoms under PRRSV infection were different among breeds^8^. In the last decade, more artificial infection experiments were conducted within different pig breeds or populations with different backgrounds, which provides a strong evidence and support for the genetic contributions to PRRSV resistance.

In 2006, a highly pathogenic PRRSV (HP-PRRSV) broke out in China, and the epidemic persisted for a long time^9^, which made pig industry in a serious deficit state. While during the outbreak of HP-PRRSV, the Tongcheng (TC) pigs, a fine local variety in central of China, displayed extremely strong resistance to PRRSV^10^. Our previous study with artificial infection showed that TC pigs were less susceptible to PRRSV than LW pigs, manifesting as less tissue lesions, less virus load in serum, lower level of IL-10 but higher level of anti-viral cytokine interferon-gamma (IFN-γ)^11^. With RNA-sequencing, we compared the transcriptome difference of PAMs between TC and LW pigs, which revealed that TC pigs may promote the extravasation and migration of leukocytes to defend against PRRSV infection^12^.

With the development and widespread application of high-throughput sequencing technology, non-coding RNAs, including microRNA (miRNA) and long intergenic non-coding RNA (lincRNA), have been gradually recognized and found to participate in numerous biological processes. Some miRNAs were reported to regulate PRRSV proliferation. For example, miR-181 could strongly inhibit PRRSV replication through binding with ORF4 and PRRSV receptor CD163^13,14^; miR-23, miR-378 and miR-505 were verified to directly target PRRSV genomic and subgenomic RNA^15^; miR-26a could up-regulate the expression level of IFN-I and ISGs^16,17^; miR-125b suppressed PRRSV replication through down-regulate NF-κB pathways^18^; while miR-373 facilitated the replication of PRRSV by negative regulation of IFN-β^19^. Except for miRNAs, growing evidence suggested that lincRNAs could serve as ceRNAs through competing with mRNAs by binding to miRNAs and an increasing number of lincRNAs were confirmed to function as regulators of immune system^20–22^. Despite of the important roles of non-coding RNAs in immune response regulation, however, our previous work as well as other reported PRRSV-related transcriptome studies was focused on the function of protein-coding genes in PRRSV infection, less is known about the expression pattern of miRNAs and lincRNAs in the process of PRRSV infection. In this study, RNA sequencing with small RNA, and total RNA of ribosomal RNA removal, from PAMs of both TC and LW pigs were performed to obtain profiles of miRNAs and lincRNAs, which we hope and believe will provide another perspective for revealing the PRRSV resistance mechanism.

## Materials and Methods

### Sample preparation and RNA isolation

Pigs used in this study were selected from our previous research^11^. The HP-PRRSV strain was PRRSV WUH3 with a dose of 10^−5^ TCID_50_ for intramuscular challenge at 3 mL per 15 kg for experimental pigs. Twelve 5-week-old piglets of Tongcheng pigs (TC, *n* = 6) and Large White pigs (LW, *n* = 6) were slaughtered on 7-days post infection and then sampled, three for control and the other three for HP-PRRSV infection, respectively. PAMs were collected by bronchioalveolar lavage from lungs, lysed with TRIzol reagent (Thermol Fisher Scientific, Waltham, MA, USA), frozen in liquid nitrogen and stored at −80 °C until RNA extraction. Tissues including heart, liver, spleen, lung, kidney, brain, testis, mesenteric lymph nodes (MLN) and inguinal lymph nodes (ILN) were also collected for RNA isolation and analysis. All animal procedures were approved by the Ethical Committee for Animal Experiments at Huazhong Agricultural University, Wuhan, China (Animal experiment approval No. HZAUSW-2013-005, 08/27/2013). The animal experiments were performed at the Laboratory Animal Center of Huazhong Agricultural University.

Total RNA was extracted according to the manufacturer’s instructions. RNA degradation and contamination were initially monitored on 1% agarose gels. RNA purity was determined using the NanoPhotometer® spectrophotometer (IMPLEN, CA, USA). Then, the quantity of RNA was measured through Qubit^®^ RNA Assay Kit in Qubit^®^ 2.0 Flurometer (Life Technologies, CA, USA). RNA integrity was assessed using the RNA Nano 6000 Assay Kit of the Agilent Bioanalyzer 2100 system (Agilent Technologies, CA, USA).

### Library construction for small RNA sequencing and data analysis

Library construction and sequencing were conducted in the Beijing Novogene Technology Co. Ltd. Company (Beijing, China). A total of 3 μg RNA per sample was used to generate a small RNA library using NEBNext^®^ Multiplex Small RNA Library Prep Set for Illumina^®^ (NEB, USA.) following manufacturer’s recommendations and index codes were added to attribute sequences to each sample. Library quality was assessed on the Agilent Bioanalyzer 2100 system. Totally, twelve libraries were constructed. After the clustering of the index-coded samples on a cBot Cluster Generation System using TruSeq SR Cluster Kit v3-cBot-HS (Illumina), the libraries were sequenced on an Illumina Hiseq. 2000 platform and 50 bp single-end reads were generated.

To guarantee the quality of subsequent analysis, raw reads were processed to obtain clean reads as described: (1) Discard reads with poly-N > 10%, or poly-A, T, C, G and low-quality reads; (2) Remove reads containing 5′adapter contaminants; (3) Filter out reads without 3′adapters or insert tags; (4) Trim 3′adaptor sequences. Length-filtered clean reads were mapped to the reference pig genome (Sscrofa 10.2) by Bowtie. Then the aligned reads were compared with pig miRNA precursors in miRBase 21.0 to find known miRNA. Custom scripts were used to obtain miRNA counts, which were later normalized by TPM (transcript per million): mapped read count × 10^6^/total read count^23^. MiRNAs with at least five reads coverage in either library of the twelve were considered to be expressed and used for the following analysis. Differential expression analysis was performed by SARTools package^24^, setting *p* value < 0.05 and Fold Change (abbreviated as FC) ≥ 2 as threshold.

Target genes of miRNAs were predicted by software miRanda^25^. Genome sequence of PRRSV-WUH3 were downloaded from NCBI (accession number: HM853673.2) and used for miRNA binding sites prediction. Meanwhile, 3′UTRs of pig mRNA from Ensembl database were extracted to find underlying host target genes of expressed miRNAs. Also, an integrated analysis between differentially expressed (DE) miRNAs and DEmRNAs^12^ was performed.

### Library construction for ribo-zero RNA sequencing and data analysis

The detailed procedure of library construction for ribo-zero RNA sequencing is described in previous publication^12^. In brief, after removal of rRNA, twelve strand-specific cDNA libraries were constructed and 100 bp paired-end raw reads were generated. Clean data were obtained by removing reads containing adapter, reads containing ploy-N and low quality reads. Clean reads were mapped to the reference pig genome (Sscrofa 10.2) by TopHat v2.0.9^26^. The aligned reads were assembled into transcripts with Cufflinks and merged together with Cuffmerge^27^. To identify lincRNA, four steps were taken: (1) transcripts with single exon or shorter than 200nt were removed; (2) coding potential score, generated with CPC software, lower than −1 was supposed to be non-coding^28^; (3) transcripts with FPKM value in all samples less than 0.01 were discarded; (4) transcripts overlapping with known gene or falling into 1 kb of any protein-coding genes were also removed^29^. Cuffdiff was used to identify DElincRNAs between groups, setting *p* value < 0.05 and |FC| ≥ 2 as threshold. DEmRNAs^12^ within 500 kb of lincRNA were regarded as the cis target genes, while DEmRNAs co-expressed with DElincRNAs were considered as trans target genes. For the predicted target genes of DEmiRNAs and DElincRNAs, gene ontology (GO) and KEGG pathway analysis were performed using DAVID (https://david.ncifcrf.gov/), considering *p* value < 0.05 as the condition of significantly enrichment.

### Construction of lincRNA-miRNA-mRNA regulatory network

Based on the sequence complementary and negative correlation between miRNA and target mRNA or target lincRNA, as well as the co-expression relationship between lincRNA and mRNA, the regulatory network of lincRNA-miRNA-mRNA was analyzed.

### qRT-PCR validation of differentially expressed miRNA and lincRNA

First strand cDNA synthesis kit (Takara, Dalian, China) was used for reverse transcription, and common qRT-PCR and stem-loop qRT-PCR were conducted respectively to determine the relative expression of lincRNAs and miRNAs. Pig U6 snRNA and *GAPDH* were used as internal control for miRNA and lincRNA, respectively. Primers used for qRT-PCR are listed in Table S1. The qRT-PCR was performed in a total volume of 10 μL containing 5 μL 2 × SYBR Green Real-time PCR Master Mix, 0.2 μM of each primer, 1 μL cDNA and 3.6 μL ddH_2_O. The qRT-PCR reaction was conducted at 95 °C for 3 min, followed by 40 cycles of 95 °C for 15 s, 60 °C for 15 s, 72 °C for 20 s. All of the reactions were run in triplicate. Relative gene expression level was calculated using the 2^−ΔΔCT^ method^30^. Student’s *t*-test was performed for data analysis, *p* value < 0.05 and *p* value < 0.01 was considered as the statistical threshold of significant difference and highly significant difference, respectively.

### Western Blot analysis

3D4-21 cells were purchased from ATCC (https://www.atcc.org/Products/All/CRL-2843.aspx) and cultured in RPMI 1640 Medium (Gibco, Thermo Fisher Scientific, New York, NY, USA) supplemented with 10% heat-inactivated fetal bovine serum (FBS) (Gibco). Cells were lysed with RIPA lysis buffer (Biosharp, Hefei, China) and centrifuged to obtain total proteins. The concentration of total proteins was measured with Bradford protein assay kit (Beyotime, Shanghai, China). After denaturation by boiling, the protein was separated on SDS-PAGE gel electrophoresis and transferred to polyvinylidene diuoride membranes. Then the membranes were blocked with skimmed milk powder, and incubated with primary antibodies (CD151 Antibody (Abcam, Cambridge, UK), GAPDH Antibody (Beyotime, Shanghai, China)) and secondary antibodies (HRP-labeled HRP-labeled Goat Anti-Rabbit IgG(H + L) (Beyotime). Finally, the membranes were visualized by ECL detection kit (Bio-Rad, Hercules, CA, USA) and imaged with ImageQuant™ LAS 4000 (GE Healthcare Life Sciences, Beijing, China). The results were analyzed by ImageJ software (https://imagej.net).

## Results

### Overview of miRNA sequencing

To reveal the expression difference of non-coding RNAs in PAMs from PRRSV infected TC and LW pigs, twelve small RNA libraries and twelve cDNA libraries were constructed and sequenced. The experiment was divided into four groups: TC_CON, TC_INF, LW_CON and LW_INF. For small RNA sequencing, the numbers of raw reads which covered at least 51.6 million bases ranged from 10M to 13M (Table 1). Q20 was above 97.89% and Q30 was above 94.70%, which indicated a high accuracy of sequencing data. Clean reads accounted for 96.92% to 98.53% of the raw reads. The length of clean reads was mainly from 20 nt to 24 nt, which is in accord with the general length range of miRNA. Of the 411 annotated mature miRNA, 208 miRNAs were transcribed in all sequenced individuals. We analyzed the first nucleotide bias of the identified miRNA, which revealed that it tended to be uracil at most cases (Fig. S1).

**Table 1:** Summary of small RNA sequencing.

Group	Individual	Raw Reads	Q30	Clean Reads	Number of Identified Known miRNA
TC_Control	TC_C1	10,725,562	95.20	10,527,099 (98.15%)	226
	TC_C2	13,295,824	95.01	13,039,082 (98.07%)	220
	TC_C3	11,928,403	94.93	11,560,645 (96.92%)	191
TC_Infection	TC_I1	10,327,597	95.16	10,175,859 (98.53%)	222
	TC_I2	10,555,502	94.70	10,290,076 (97.49%)	228
	TC_I3	12,071,262	94.95	11,874,927 (98.37%)	208
LW_Control	LW_C1	12,840,964	95.12	12,611,542 (98.21%)	218
	LW_C2	12,615,677	96.00	12,427,400 (98.51%)	222
	LW_C3	11,830,355	96.29	11,604,358 (98.09%)	218
LW_Infection	LW_I1	13,432,927	95.04	13,099,988 (97.52%)	229
	LW_I2	11,741,299	95.62	11,550,964 (98.38%)	226
	LW_I3	11,019,788	95.94	10,829,267 (98.27%)	220

### Identification of DEmiRNAs and qRT-PCR validation

**Figure 1:**
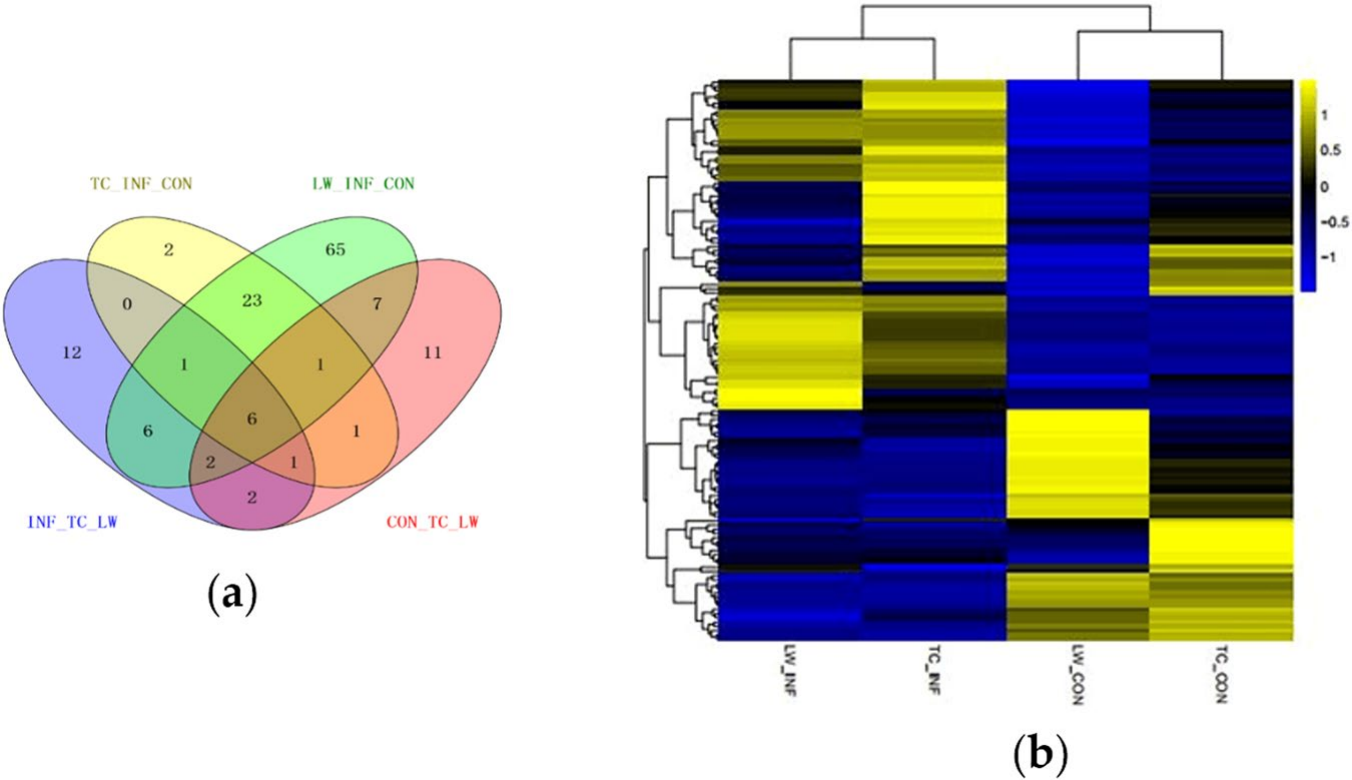
Analysis of DEmiRNAs. (**a**) Venn diagram for the distribution of DEmiRNAs in the four comparisons. (**b**) Heatmap showing the expression level for DEmiRNAs. MicroRNAs shown in yellow means higher expression and blue means lower expression.

Setting *p* value < 0.05 and |FC| ≥ 2 as the criteria for screening DEmiRNAs, 67 up-regulated and 44 down-regulated miRNAs were identified to be differentially expressed after PRRSV infection in LW pigs. Also, compared with TC_Control group, 23 up-regulated and 12 down-regulated miRNAs were identified in TC_Infection group (Table S2). Additionally, there were 31 and 30 miRNAs differentially expressed in the control and infection groups between these two breeds, respectively (Table S2; Fig. 1a). It is worth noting that miR-451, miR-486, miR-199b-3p, miR-199a-3p, miR-199b-5p and miR-31 were differentially expressed in all the four paired-comparisons (Table S2): CON_TC vs CON_LW, INF_TC vs INF_LW, LW_CON vs LW_INF and TC_CON vs TC_INF. Heatmap for all DEmiRNAs in the four groups revealed that TC_CON and LW_CON displayed similar expression pattern and clustered together, and the two infection groups clustered together. However, within one cluster, no matter control or infection group, some miRNAs displayed extreme expression difference between the two breeds (Fig. 1b). miR-378, miR-378b-3p, miR-582 and miR-7135-3p were specifically down-regulated in PAMs of TC pigs, while there were 80 specific DEmiRNAs in PAMs of LW pigs.

**Figure 2:**
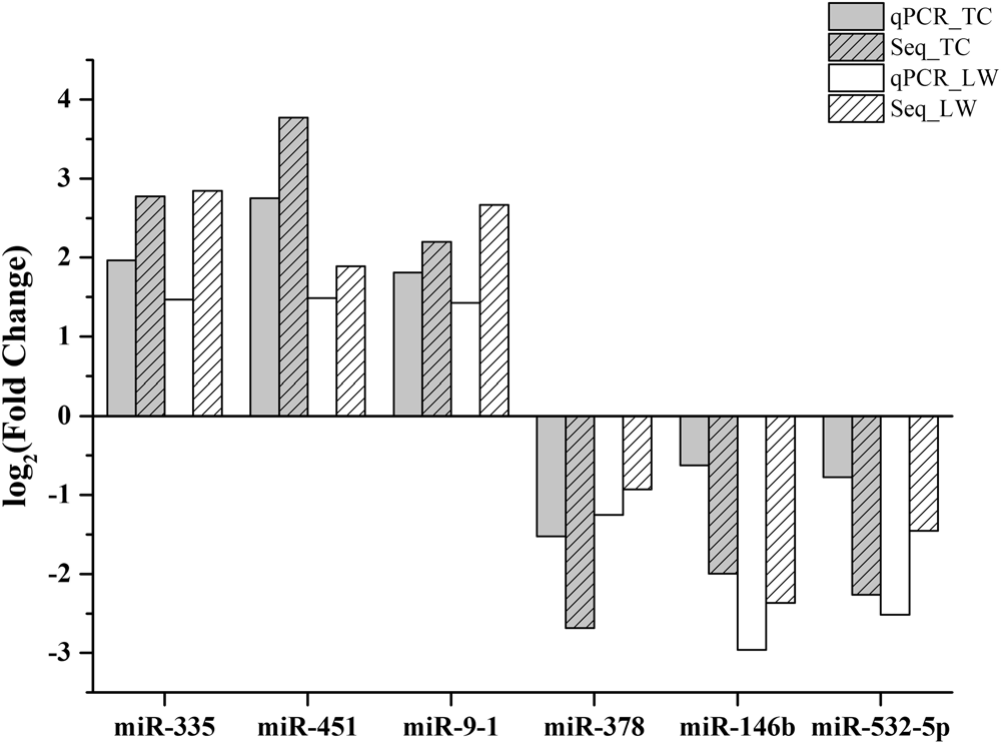
Log_2_(FC) obtained from stem-loop qRT-PCR and small RNA-seq data. *x*-axis is the name of selected DEmiRNA and *y*-axis is the value of log_2_(FC).

To validate the analysis results, six DEmiRNAs (miR-146b, miR-335, miR-378, miR-451, miR-532-5p and miR-9-1) were randomly selected for stem-loop qRT-PCR assay. The results indicated that trends of relative expression of qRT-PCR were consistent with small RNA-seq (Fig. 2).

### Functional analysis of the target genes of DEmiRNAs

**Figure 3:**
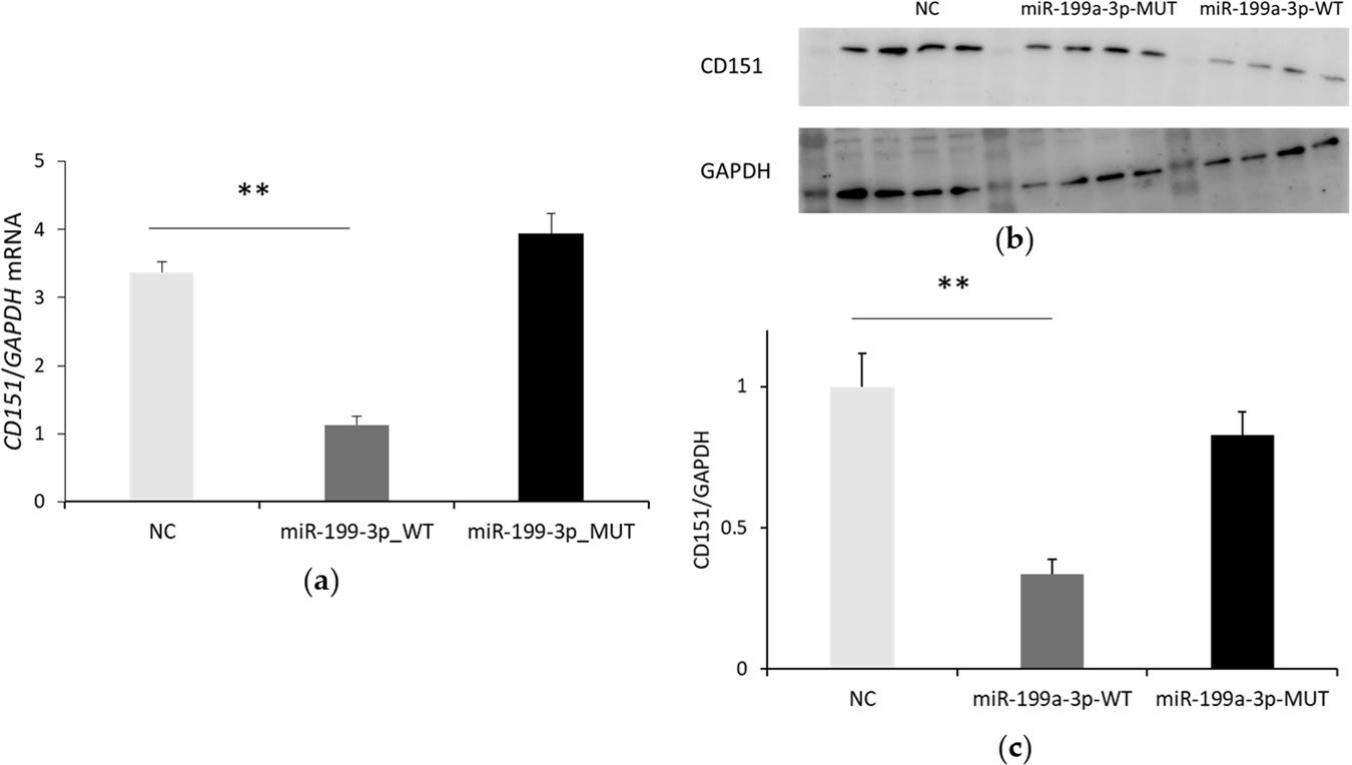
miR-199a-3p could inhibit the expression of CD151. Endogenous CD151 mRNA (**a**) and protein (**b**) expression under the condition of miR-199a-3p overexpression (NC, negative control; MUT, seed region mutated type; WT, wild type) was detected by qRT-PCR and Western blot, respectively; (**c**) Relative expression of CD151 normalized to GAPDH. Every four bands were a replicate, and this experiment was repeated for three times. Student’s *t*-test was used to compare the differences between miR-199-3p-MUT/WT and NC, and one star (*) represented *p* <0.05.

miRanda was used to predict target genes of DEmiRNAs against PRRSV genome, PRRSV receptor and other host genes. Of the shared DEmiRNAs before and after infection in the two breeds, 23 miRNAs could bind to PRRSV genome (Table S3). Of which, miR-1343, miR-296-3p, miR-199a-3p and miR-34c could target PRRSV receptor CD151; miR-181a and miR-181b could target CD163 and miR-34c could target CD209. They were differentially expressed between control and infection group in both breeds (Table S2), which indicated that these common DEmiRNAs might play a key role in the regulation of PRRSV infection in pigs. In addition, four specific DEmiRNAs in TC pigs (miR-378, miR-378b-3p, miR-7135-3p and miR-582) were predicted to have ten binding sites on PRRSV genome. Then miR-199a-3p was randomly selected to validate the result of bioinformatics analysis. As shown in Fig. 3, over-expression of miR-199a-3p by mimics downregulated the expression of CD151.

**Figure 4:**
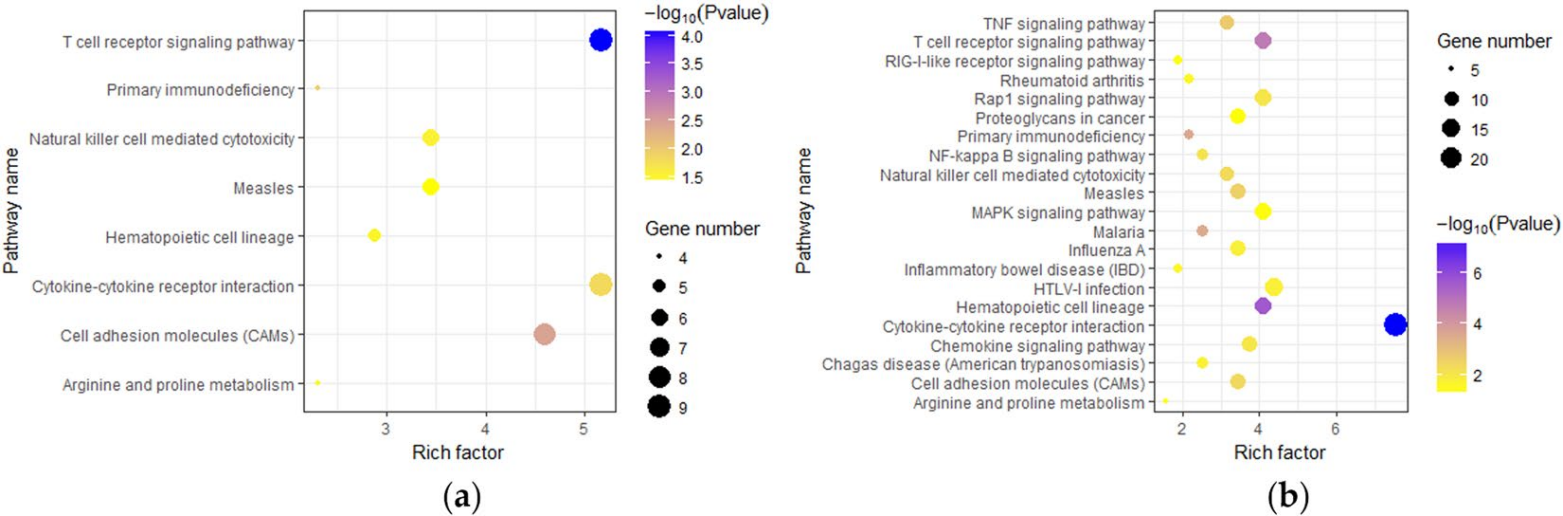
Enriched KEGG Pathway for targets of down-regulated DEmiRNAs responded to PRRSV. (**a**) downregulated miRNA in TC pigs; (**b**) downregulated miRNA in LW pigs.

We further predicted the target genes of DEmiRNAs. Interestingly, the target genes of down-regulated miRNAs were enriched in apoptosis process, inflammatory response, T cell receptor signaling pathways, natural killer cell mediated cytotoxicity and other immune-related process (Table 2; Fig. 4; Table S4). After analysis on the target genes, multiple immunosuppressive receptor/ligand genes were found. In LW pigs, specifically down-regulated miR-185 (log_2_ FC = −1.09, *p* value = 1.50E-03) could target specifically up-regulated *Siglec5* (log_2_ FC = 1.70, *p* value = 5.00E-05); miR-145-5p (log_2_ FC = −1.67, *p* value = 7.91E-04), which had an up-regulated trend in TC pigs, could target two DEmRNAs: *CTLA4* (log_2_ FC = 4.22, *p* value = 5.00E-05) and *Tim-3* (log_2_ FC = 3.43, *p* value = 5.00E-05). We inferred that the immune system of LW pigs was suppressed at a higher degree post infection.

**Table 2:** The top 10 GO biological process terms for targets of all down-regulated miRNAs in TC and LW pigs.

TC_GO Terms	No. of Genes	LW_GO Terms	No. of Genes
apoptotic process	16	signal transduction	40
immune response	14	immune response	34
oxidation-reduction process	12	inflammatory response	31
defense response to virus	10	regulation of transcription from RNA pol II promoter	29
cell surface receptor signaling pathway	9	apoptotic process	27
inflammatory response	9	cell surface receptor signaling pathway	19
T cell receptor signaling pathway	8	positive regulation of transcription, DNA-templated	19
Angiogenesis	7	positive regulation of cell proliferation	18
protein dephosphorylation	6	cell adhesion	17
positive regulation of cytosolic calcium ion concentration	6	positive regulation of GTPase activity	17
positive regulation of I-κB kinase/NF-κB signaling	6	cellular response to LPS	16
response to lipopolysaccharide	6	response to virus	14
cell migration	6	response to LPS	14
T cell activation	5	T cell receptor signaling pathway	13
B cell receptor signaling pathway	5	cell-cell signaling	13

### Identification and qRT-PCR validation of differentially expressed lincRNAs

**Figure 5:**
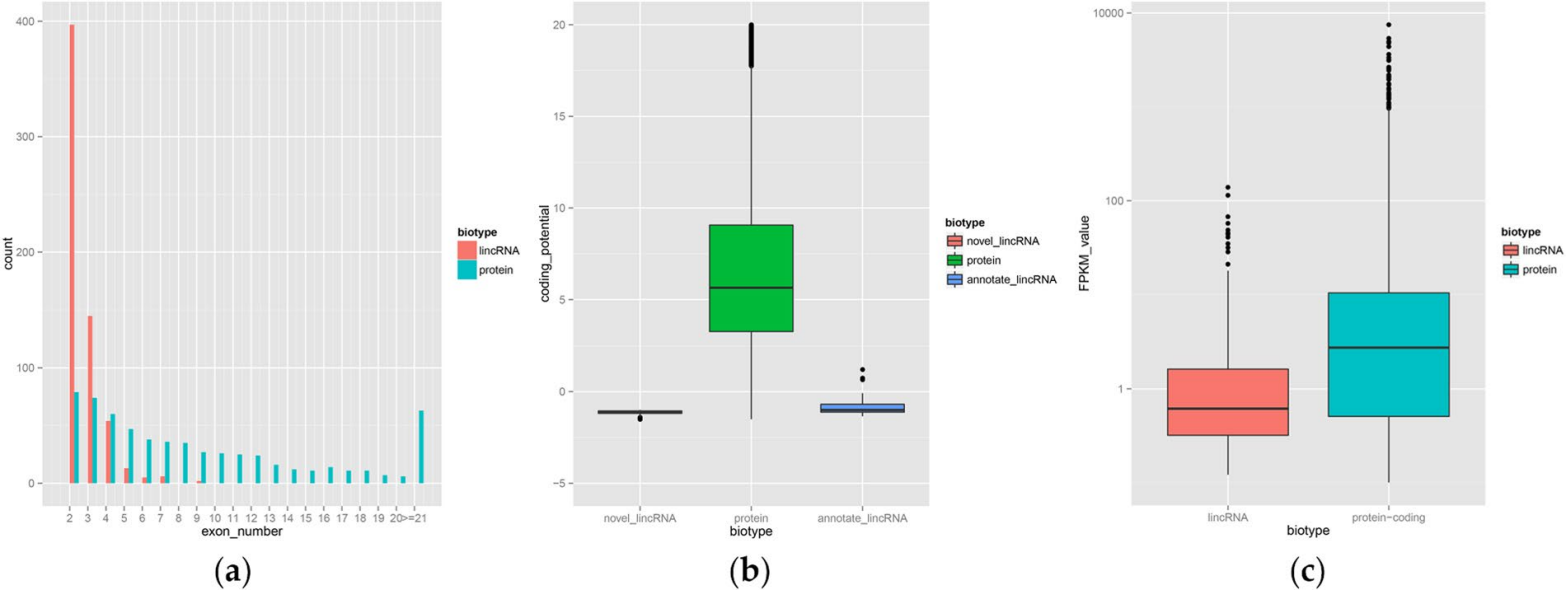
Characteristics of lincRNAs. (**a**) Comparison of exon number between the novel lincRNA and protein_coding gene. (**b**) Comparison of coding potential among the identified lincRNA and the annotated lincRNA and protein_coding gene. (**c**) Comparison of expression level between lincRNA and protein_coding gene.

Overview of ribo-zero RNA sequencing data were described in another publication^12^. After series of rigorous screening, 616 lincRNA transcripts were identified eventually, which were distributed on each chromosome except chromosome Y. The average length of these lincRNA transcripts is 2709 nt and more than 60% of the lincRNAs contain only two exons. Compared with protein-coding genes, the identified lincRNA has fewer exon number, lower coding potential and lower expression level (Fig. 5).

**Figure 6:**
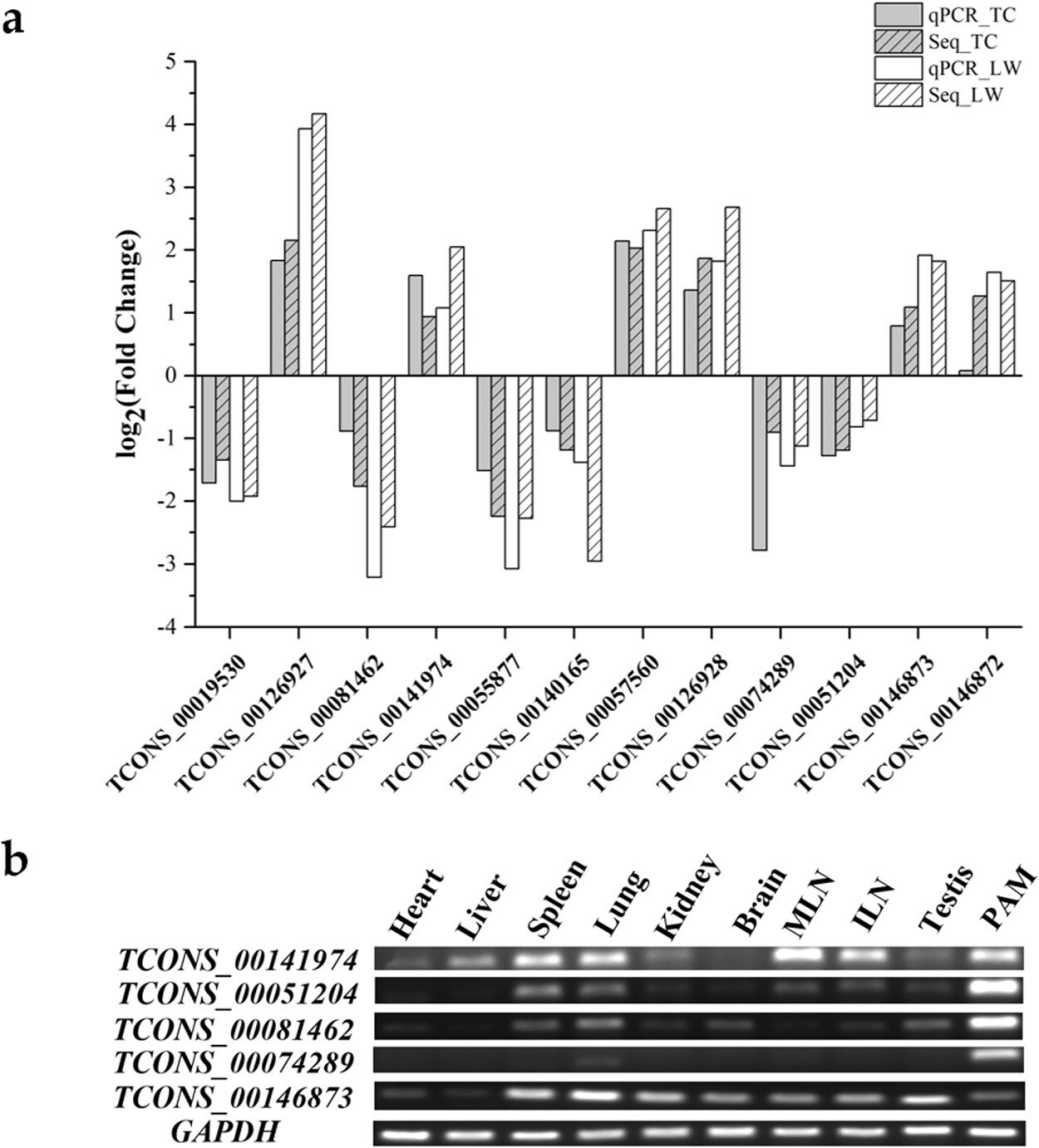
(**a**) Log2 (FC) obtained from qRT-PCR and RNA-seq data. X-axis is the name of selected DElincRNA and Y-axis is the value of log2 (FC); (**b**) Tissue/cell expression analysis of five lincRNAs. MLN represents for mesenteric lymph nodes and ILN represents for inguinal lymph nodes.

Compared with TC control group, there were 11 significantly down-regulated and 19 up-regulated lincRNAs in TC infection group. Also, 29 lincRNAs and 19 lincRNAs were found to be down-regulated and up-regulated in LW infection group, respectively (Table S5). 11 DElincRNAs were randomly selected to validate through qRT-PCR. The results showed that the relative expression of lincRNAs was consistent with RNA-seq analysis result (Fig. 6a). Additionally, the expression level of 5 DElincRNAs was detected in ten tissues by RT-PCR, which revealed that the selected DElincRNAs expressed highly in immune system except *TCONS_00146873* (Fig. 6b).

### Functional annotation of the target genes of DElincRNAs

To explore potential cis-regulatory lincRNAs, DEmRNAs within 500 kb upstream and downstream of DElincRNAs were analysed^31^. Totally, 150 DEmRNAs were found and they were enriched in regulation of signal transduction and reproduction-associated biological processes (Table S6). Based on trans-acting, Pearson Correlation Coefficient of DElincRNAs and DEmRNAs was calculated. Result showed that 915 DEmRNAs co-expressed with lincRNAs were found, which were significantly enriched in multiple immune-related biological processes (Table 3). Target genes of DElincRNAs between control and infection group in both breeds were enriched in the similar biological processes and KEGG pathways.

**Table 3:** GO biological process terms and KEGG pathway for DEmRNAs co-expressed with DElincRNAs.

Item	Name	No. of Genes
GO BP terms	immune,defense and inflammatory response	79
	regulation of cell death	55
	negative regulation of apoptosis	31
	regulation of cell(lymphocyte) activation	23
	positive regulation of immune system process	22
	regulation of inflammatory response	12
KEGG Pathway	Cytokine-cytokine receptor interaction	24
	Chemokine signaling pathway	17
	Cell adhesion molecules (CAMs)	15
	T cell receptor signaling pathway	12
	Hematopoietic cell lineage	11
	Leukocyte transendothelial migration	11
	Lysosome	10
	Graft-versus-host disease	6
	Allograft rejection	6
	Autoimmune thyroid disease	6
	Primary immunodeficiency	5

### Construction of lincRNA-miRNA-mRNA regulatory network

**Figure 7:**
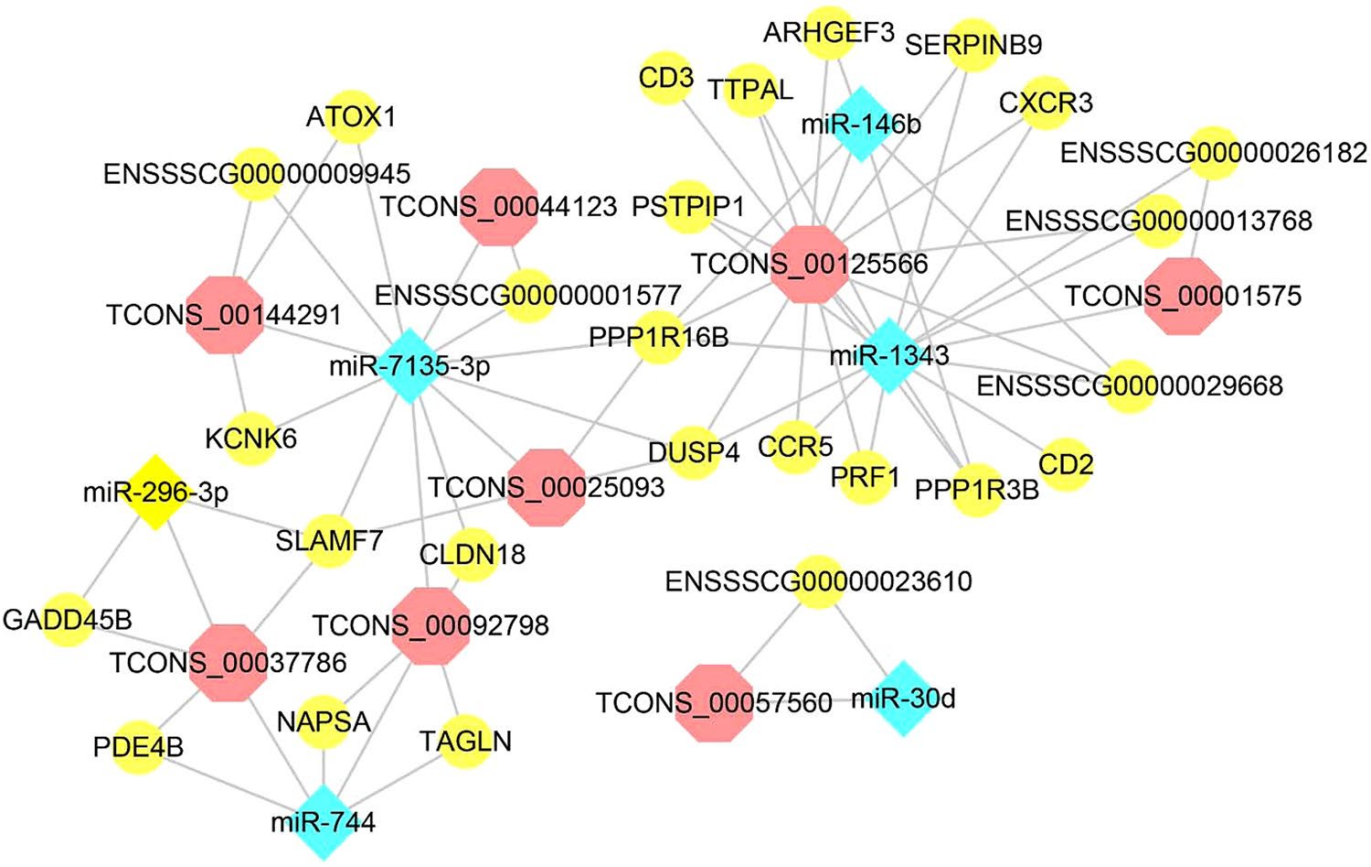
Regulatory network of lincRNA-miRNA-mRNA.

As down-regulated miRNAs closely related to immunity, they were used to do the following analysis. Based on the sequence complementarity and negative correlation between miRNA and its targets, as well as the co-expression relationship between lincRNA and mRNA, the regulatory network of lincRNA-miRNA-mRNA was analysed (Fig. 7). The results showed that lincRNA *TCONS_00125566* (TC: log_2_ FC = 1.85, *p* value = 0.03; LW: log_2_ FC = 2.04, *p* value = 0.01) was in the hub of the regulatory network. It was forecast to regulate the expression of *CCR5*, *DUSP4*, *CXCR3* and other genes through binding with miR-1343 competitively, which implyed that *TCONS_00125566* and *miR-1343* (TC: log_2_ FC = −1.78, *p* value = 0.03; LW: log_2_ FC = −1.76, *p* value = 5.89E-06) could play a crucial part in the regulation of PRRSV-host interaction. LincRNA *TCONS_00037786* (TC: log_2_ FC = 1.49, *p* value = 0.03; LW: log_2_ FC = 2.07, *p* value = 2.95E-03) could target *GADD45B* and *SLAMF7* through binding with miR-296-3p (TC: log_2_ FC = −2.99, *p* value = 1.35E-03; LW: log_2_ FC = −2.95, *p* value = 3.00E-11).

## Discussion

PRRS has been pandemic for over 30 years, but it’s still one of the main enemies of large-scale pig farms. Clinical prevention is difficult due to its high variability and the pressure of immune selection. Therefore, it is particularly important to enhance the genetic resistance to PRRSV and further improve the genetic structure. In this study, small RNA-seq and ribo-zero RNA-seq were performed to study the regulation of miRNA and lincRNA in the interaction between virus and host, which not only provides a new facet to investigate the differences of disease resistance of TC and LW pigs, but also lays foundation for further studying the antiviral function of miRNA and lincRNA. As a result, we identified some miRNAs and lincRNAs which may play important roles in PRRSV defending in two breeds, including miR-181, miR-296-3p, miR-744, miR-185, let-7c, miR-145-5p, miR-328, etc., as well as *TCONS_00074289*, *TCONS_00037786*, *TCONS_00125566* and so on.

As an important regulatory factor, miRNAs participate in diverse biological process, such as development, metabolism, cell proliferation and apoptosis, also in the intricate networks of host-pathogen interactions and innate immunity. In accordance with previous research, miR-181, validated to inhibit PRRSV replication^13,14^, was up-regulated post infection compared with control groups in both breeds. miR-296-3p and miR-744 was decreased more than 5-fold in both breeds, and they could target *LDOC1 (*up-regulated post-infection), which served as a negative regulator of NF-κB^32^ and was involved in anti-inflammatory response in both two breeds. Interestingly, miR-451 was up-regulated in both breeds, while it was expressed higher in TC pigs, even 17 times to LW infection group. Previous research indicated that miR-451 could inhibit the expression of pro-inflammatory factors^33^. Thus, its higher expression level probably contributed to relatively weakened tissue inflammation in TC pigs. It is also well known that PRRSV is an immunosuppressive disease, and IL-10 is a vital immunosuppressive factor during PRRSV infection^34^. In the current study, miR-185 and let-7c, which were specifically down-regulated in LW pigs, targeted *SIGLEC5* that was up-regulated during PRRSV infection. Previous study showed that mouse SIGLEC5 enhanced IL-10 production while inhibiting TNF-α production in macrophages^35^. As a member of Siglec family, SIGLEC5 could facilitate the escape of pathogenic organisms from the control of the natural immune system^36^. Thus, LW pigs with higher expression of SIGLEC5 and IL10 manifested a state of immunosuppression. In addition, the specifically down-regulated miR-145-5p in LW pigs could target Cytotoxic T-lymphocyte associated protein 4 (CTLA-4), which had a significant lower expression in TC infection group than that in LW infection group. CTLA-4, as a negative regulator of T-cell activation, could reduce response to antigen^37^. Besides, miR-328, specifically up-regulated in LW pigs, could target programmed death ligand-1 (PD-L1), which was up-regulated in LW infection group compared with TC infection group. Research showed that increased PD-L1 expression on antigen presenting cells (APCs) could aid in virus survival and decrease T-cell activity^38^. *TIM-3*, which could inhibit Th1 cells’ immune activity by mediating apoptosis and thus inducing immune suppression, might be targeted by miR-362 and miR-365-3p. To sum up, the above mentioned DEmiRNAs were specifically down-regulated in LW pigs, while all of their target genes were up-regulated in LW pigs. Interestingly, these target genes together with IL-10 participated in immunosuppression, which indicated that some DEmiRNAs were involved in the regulation of immunosuppression in LW pigs. As a common DEmiRNAs of two breeds, miR-199 was effective in targeting and regulating HBV (Hepatitis B virus), thus curing the disease caused by HBV^39^. In this study, we found miR-199a-3p could inhibit CD151 expression at protein level. Since CD151 serves as the receptor of PRRSV, this would be an appropriate way to prevent PRRSV by miRNAs through modulating their targeted genes.

Apart from miRNAs, lincRNA is also an indispensable part of transcriptome. However, pig lincRNAs of immune system have been rarely reported. In the current study, we identified 616 differentially expressed lincRNA transcripts in PAMs, between control and infection group within the each pig breed or between control groups of the two pig breeds.

Co-expression analysis based on Pearson correlation coefficient identified many DEmRNAs, which were involved in immune-related process and pathway, including inflammatory response, apoptosis, cytokine-cytokine receptor interaction and so on. The regulation of these DEmRNAs by lincRNA was mostly mediated by trans-acting, which was consistent with a previous research^40^. Interestingly, one DElincRNA—*TCONS_00074289*—was expressed only in PAMs and lung, which indicated its function in the development of lung and PAMs. Recent reports have suggested that lincRNAs can potentially interact with other classes of non-coding RNAs including miRNAs and then regulate the expression of mRNA^41^.

In this study, we systematically analyzed the complex effects of the interactions between miRNAs and their target genes and provided lncRNA-miRNA-mRNA networks. *TCONS_00037786* and *TCONS_00125566* were recognised as important competing endogenous RNA. Of the target genes, *GADD45B* was an anti-apoptosis factor^42^; *SLAMF7* could inhibit the production of proinflammatory cytokines^43^; Belong to chemokine receptor family*, CXCR3* and *CCR5* were involved in inflammatory response; *DUSP4* is closely related with cell proliferation, differentiation and apoptosis through negatively regulating MAPK pathway^44^. Therefore, we speculated that these two lincRNAs together with miR-296-3p and miR-1343 took part in the regulation of inflammination and apoptosis in pigs, which of course need further experimental validation.

In summary, miRNAs were involved in PRRSV defending, and due to the difference of genetic background, some of them displayed specific expression pattern in one breed, and also conbined with lincRNAs to modulate physiological process such as anti-virus and regulation for host immune, which probably provides a new evidence for the genetic contributions to PRRSV resistance.

## Conclusions

In this study, the expression and regulation of miRNA and lincRNA were analyzed in TC and LW pigs responded to PRRSV. Down-regulated miRNAs were involved in the regulation of PRRSV proliferation by participating in immune-related biological processes and pathway. Also, miRNA could target immunosuppressive receptor and ligand genes, leading to a stronger degree of immunosuppression in Large White pigs. Moreover, network interaction analysis was performed and some functional non-coding RNAs were found. This study lays the foundation for exploring the interaction mechanism between host and PRRSV and further revealing the disease resistance mechanisms of Tongcheng pigs.

## Electronic supplementary material

Dataset1

Dataset2

Dataset3

Dataset4

Dataset5

Dataset6

Dataset7

## References

[1] Vande Van Gorp H, Van Breedam W, Delputte PL, Nauwynck HJ (2008). Sialoadhesin CD163 join forces during entry of the porcine reproductive and respiratory syndrome virus. J. Gen Virol..

[2] Jusa ER, Inaba Y, Kouno M, Hirose O (1997). Effect of heparin on infection of cells by porcine reproductive and respiratory syndrome virus. Am. J. Vet. Res..

[3] Vanderheijden N (2003). Involvement of sialoadhesin in entry of porcine reproductive and respiratory syndrome virus into porcine alveolar macrophages. J. Virol..

[4] Kim JK, Fahad AM, Shanmukhappa K, Kapil S (2006). Defining the cellular target(s) of porcine reproductive and respiratory syndrome virus blocking monoclonal antibody 7G10. J. Virol..

[5] Shanmukhappa K, Kim JK, Kapil S (2007). Role of CD151, a tetraspanin, in porcine reproductive and respiratory syndrome virus infection. Virol J..

[6] Huang YW, Dryman BA, Li W, Meng XJ (2009). Porcine DC-SIGN: Molecular cloning, gene structure, tissue distribution and binding characteristics. Dev. Comp. Immunol..

[7] Bautista EM, Goyal SM, Yoon IJ, Joo HS, Collins JE (1993). Comparison of porcine alveolar macrophages and CL 2621 for the detection of porcine reproductive and respiratory syndrome (PRRS) virus and anti-PRRS antibody. J. Vet. Diag. Invest..

[8] Halbur PG (1998). Differences in susceptibility of Duroc, Hampshire, and Meishan pigs to infection with a high virulence strain (VR2385) of porcine reproductive and respiratory syndrome virus (PRRSV). J. Anim. Breed. Genet..

[9] Tian K (2007). Emergence of fatal PRRSV variants: Unparalleled outbreaks of atypical PRRS in china and molecular dissection of the unique hallmark. PloS ONE.

[10] Zhou P (2011). Molecular characterization of transcriptome-wide interactions between highly pathogenic porcine reproductive and respiratory syndrome virus and porcine alveolar macrophages *in vivo*. Int. J. Biol. Sci..

[11] Liang W (2016). Differences of immune responses between Tongcheng (Chinese local breed) and Large White pigs after artificial infection with highly pathogenic porcine reproductive and respiratory syndrome virus. Virus Res..

[12] Liang W (2017). Transcriptome differences in porcine alveolar macrophages from tongcheng and large white pigs in response to highly pathogenic porcine reproductive and respiratory syndrome virus (PRRSV) infection. Int. J. Mol. Sci..

[13] Guo XK (2013). Increasing expression of microRNA 181 inhibits porcine reproductive and respiratory syndrome virus replication and has implications for controlling virus infection. J. Virol..

[14] Gao L (2013). MicroRNA 181 suppresses porcine reproductive and respiratory syndrome virus (PRRSV) infection by targeting PRRSV receptor CD163. J. Virol..

[15] Zhang Q (2014). MicroRNA-23 inhibits PRRSV replication by directly targeting PRRSV RNA and possibly by upregulating type i interferons. Virology.

[16] Li L (2015). Host miR-26a suppresses replication of porcine reproductive and respiratory syndrome virus by upregulating type I interferons. Virus Res..

[17] Jia X (2015). Cellular microRNA miR-26a suppresses replication of porcine reproductive and respiratory syndrome virus by activating innate antiviral immunity. Scientific reports..

[18] Wang D (2013). miR-125b reduces porcine reproductive and respiratory syndrome virus replication by negatively regulating the NF-κB pathway. PloS ONE.

[19] Chen J (2017). MicroRNA 373 facilitates the replication of porcine reproductive and respiratory syndrome virus by its negative regulation of type I interferon induction. J. Virol..

[20] Cesana M (2011). A long noncoding RNA controls muscle differentiation by functioning as a competing endogenous RNA. Cell.

[21] Li ZH (2014). The long noncoding RNA THRIL regulates TNFα expression through its interaction with hnRNPL. P. Natl. Acad. Sci..

[22] Guttman M (2009). Chromatin signature reveals over a thousand highly conserved large non-coding RNAs in mammals. Nature.

[23] Zhou LA (2010). Integrated profiling of microRNAs and mRNAs: MicroRNAs located on Xq27.3 associate with clear cell renal cell carcinoma. PloS ONE.

[24] Varet H, Brillet-Gueguen L, Coppee JY, Dillies MA (2016). SARTools: A DESeq. 2- and EdgeR-based R pipeline for comprehensive differential analysis of RNA-Seq data. PloS ONE.

[25] Enright AJ (2003). MicroRNA targets in *Drosophila*. Genome Biol..

[26] Kim D (2013). TopHat2: Accurate alignment of transcriptomes in the presence of insertions, deletions and gene fusions. Genome Biol..

[27] Trapnell C (2010). Transcript assembly and quantification by RNA-Seq reveals unannotated transcripts and isoform switching during cell differentiation. Nat. Biotechnol..

[28] Kong L (2007). CPC: Assess the protein-coding potential of transcripts using sequence features and support vector machine. Nucleic acids Res..

[29] Orom UA (2010). Long noncoding RNAs with enhancer-like function in human cells. Cell..

[30] Livak KJ, Schmittgen TD (2001). Analysis of relative gene expression data using real-time quantitative PCR and the 2^−ΔΔc(t)^ method. Methods.

[31] Zhou ZY (2014). Genome-wide identification of long intergenic noncoding RNA genes and their potential association with domestication in pigs. Genome Biol. Evol..

[32] Nagasaki K (2003). Leucine-zipper protein, LDOC1, inhibits NF-κB activation and sensitizes pancreatic cancer cells to apoptosis. Int. J. Cancer.

[33] Rosenberger CM (2012). miR-451 regulates dendritic cell cytokine responses to influenza infection. J. Immunol..

[34] Song S (2013). Porcine reproductive and respiratory syndrome virus infection activates IL-10 production through NF-κB and p38 MAPK pathways in porcine alveolar macrophages. Devel. Comp. Immunol..

[35] Ando M, Tu W, Nishijima K, Iijima S (2008). Siglec-9 enhances iL-10 production in macrophages via tyrosine-based motifs. Biochem. Biophys. Res. Commun..

[36] Carlin AF (2009). Group b streptococcus suppression of phagocyte functions by protein-mediated engagement of human Siglec-5. J. Exp. Med..

[37] Khamri, W. *et al*. Increased expression of CTLA4 by T cells, induced by B7 in sera, reduces adaptive immunity in patients with acute liver failure. *Gastroenterology* (2017). 10.1053/j.gastro.2017.03.023 PMC5516432 28363639

[38] Latchman YE (2004). PD-L1-deficient mice show that PD-L1 on T cells, antigen-presenting cells, and host tissues negatively regulates Tcells. Proc. Natl. Acad. Sci..

[39] Lamontagne J, Steel LF, Bouchard MJ (2015). Hepatitis B virus and microRNAs: Complex interactions affecting hepatitis B virus replication and hepatitis B virus-associated diseases. World J. Gastroenterol..

[40] Guttman M (2011). LincRNAs act in the circuitry controlling pluripotency and differentiation. Nature.

[41] Salmena L, Poliseno L, Tay Y, Kats L, Pandolfi PP (2011). A ceRNA hypothesis: The Rosetta Stone of a hidden RNA language?. Cell..

[42] Engelmann A, Speidel D, Bornkamm GW, Deppert W, Stocking C (2008). Gadd45β is a pro-survival factor associated with stress-resistant tumors. Oncogene.

[43] Kim JR, Horton NC, Mathew SO, Mathew PA (2013). CS1 (SLAMF7) inhibits production of proinflammatory cytokines by activated monocytes. Inflamm. Res..

[44] Balko JM (2013). Activation of MAPK pathways due to DUSP4 loss promotes cancer stem cell-like phenotypes in basal-like breast cancer. Cancer Res..

