# Supplementary material for: Identification of Differentially Expressed Non-coding RNA in Porcine Alveolar Macrophages from Tongcheng and Large White Pigs Responded to PRRSV
Source: Sci Rep. 2018 Oct 23;8:15621. doi: 10.1038/s41598-018-33891-0 (PMC6199292; doi:10.1038/s41598-018-33891-0)
Supplement: Supplementary file 1 — Dataset1 [file 41598_2018_33891_MOESM1_ESM.doc]

Identification of Differentially Expressed Non-coding RNA in Porcine Alveolar Macrophages from Tongcheng and Large White Pigs Responded to PRRSV

Yueran Zhen ^1, 2^, Fengqing Wang ^1, 2^, Wan Liang ^1,2^, Jianjian Liu ^1,2^, Guoli Gao ^1,2^, Yan Wang ^1,2^, Xuewen Xu ^1,2^, Qiuju Su ^1,2^, Qingde Zhang ^3^, and Bang Liu ^1,2^,*

^1^ Key Laboratory of Agricultural Animal Genetics, Breeding and Reproduction of Ministry of Education, Key Laboratory of Pig Genetics and Breeding of Ministry of Agriculture & College of Animal Science and Technology, Huazhong Agricultural University, Wuhan 430070, China; zhenyueran@webmail.hzau.edu.cn (Y.Z.); 1058268233@qq.com (F.W.); liangwan521521@163.com (W.L.); liujianjian@webmail.hzau.edu.cn (J.L.); 1114215199@qq.com(G.G.); xuewen_xu@mail.hzau.edu.cn (X.X.); 365402515@qq.com (Q.S.)

^2^ The Cooperative Innovation Center for Sustainable Pig Production, Wuhan 430070, China

^3^ Laboratory Animal Center, College of Animal Science and Technology & Veterinary Medicine, Huazhong Agricultural University, Wuhan 430070, China; qdzhang@mail.hzau.edu.cn

* Correspondence: liubang@mail.hzau.edu.cn; Tel.: +86-027-8738-2290 (B.L.)


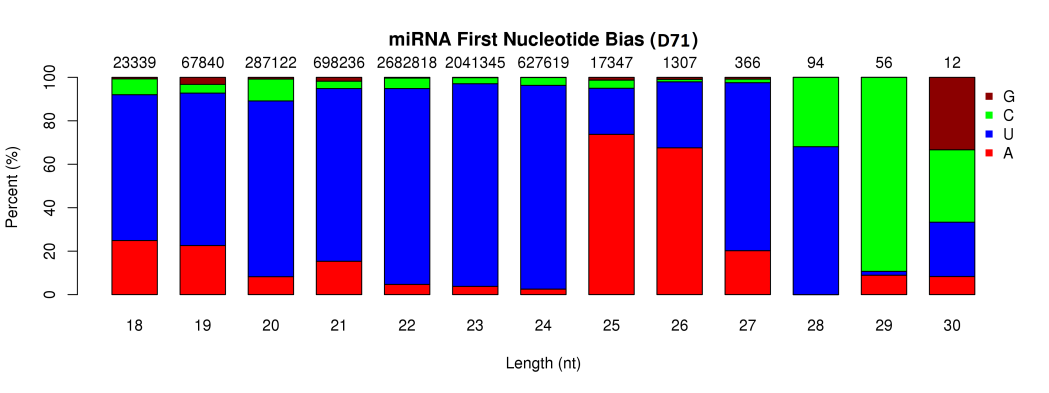


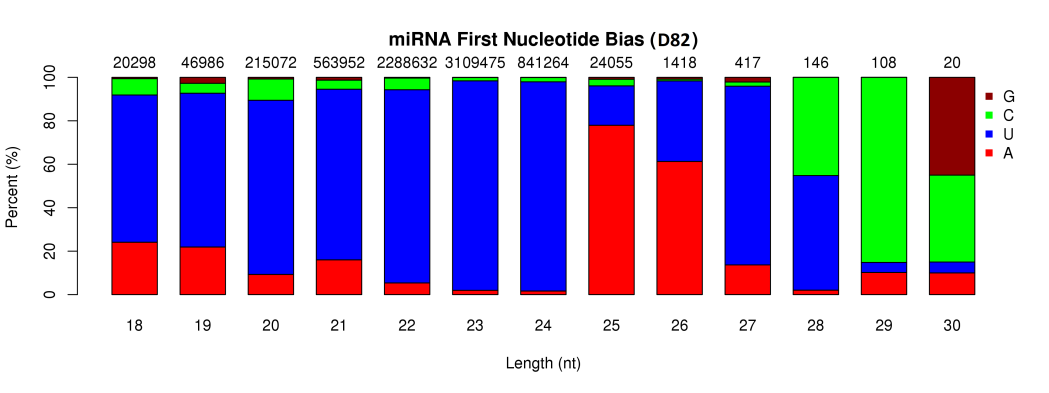


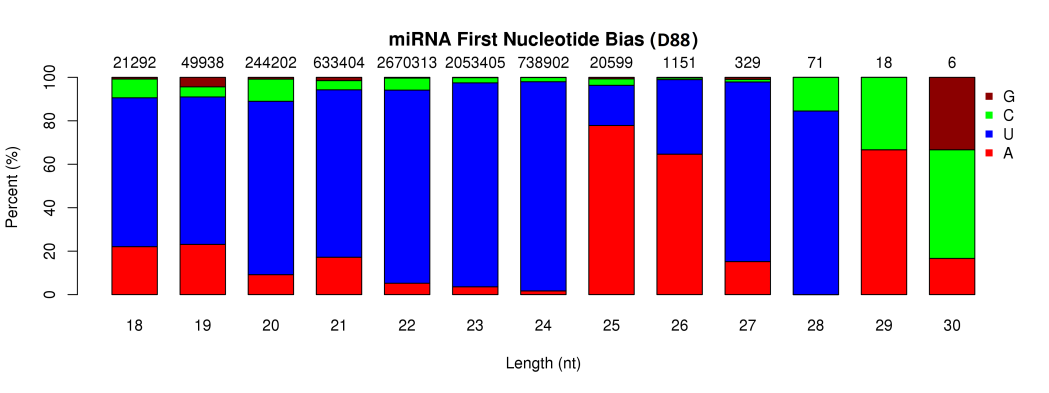


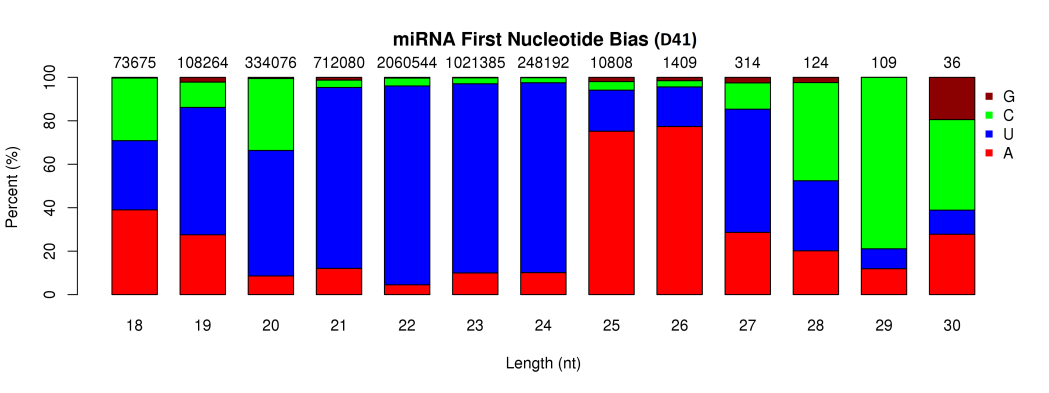


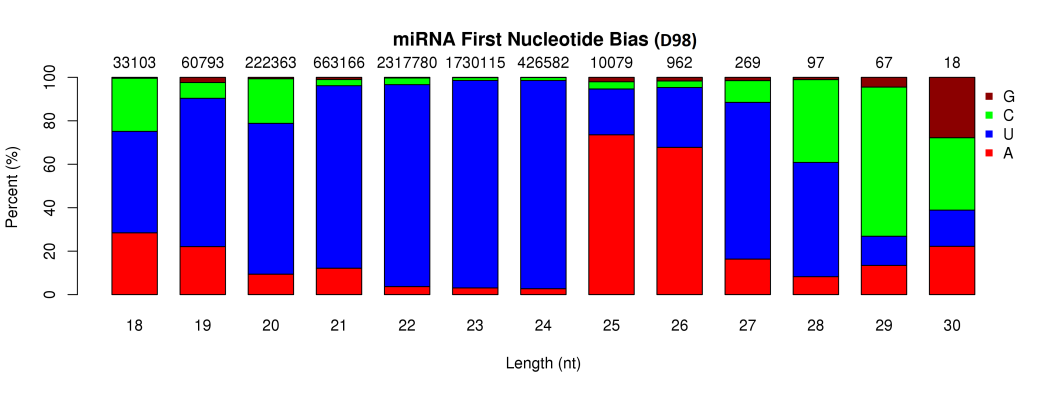


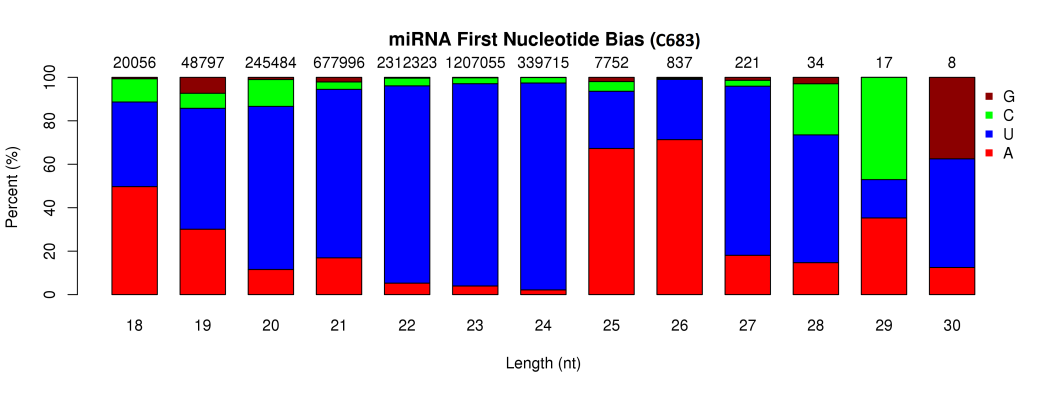

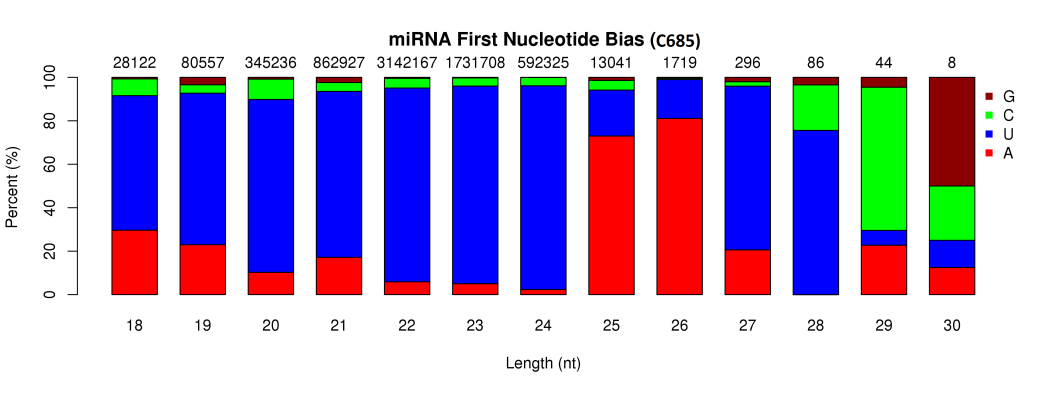

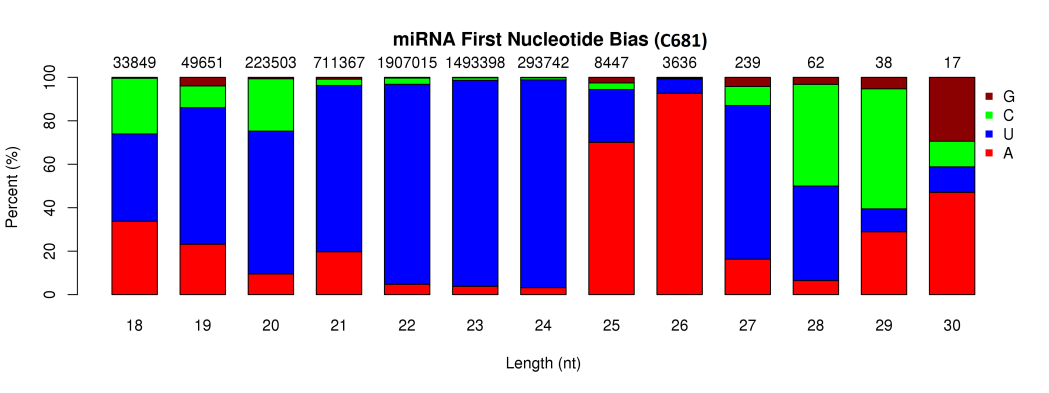


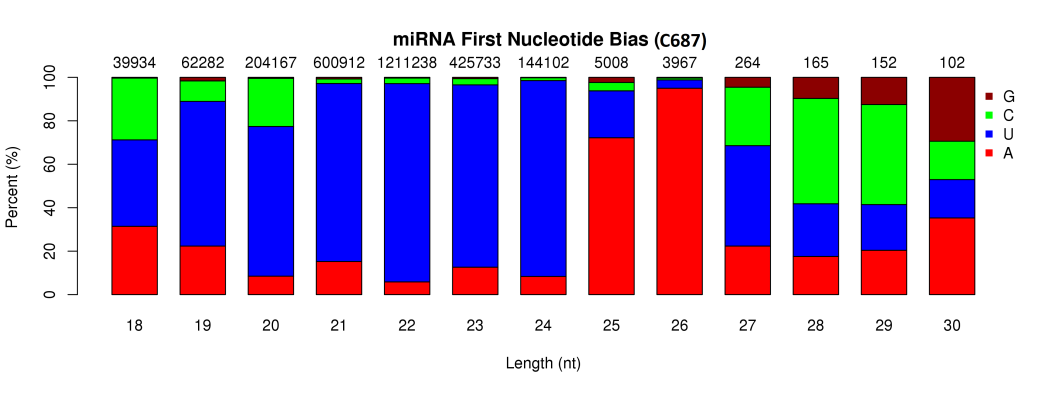


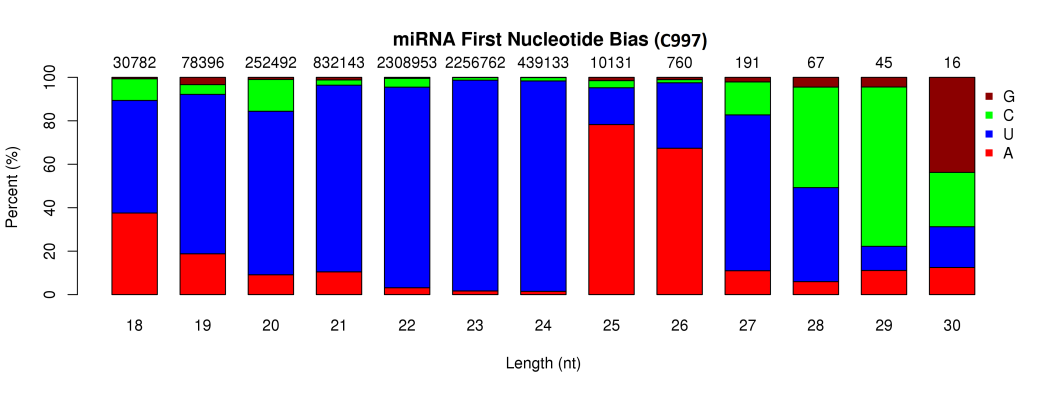


**Supplementary Figure S1.** Base preferences of the first site for the known miRNA in the 12 libraries. The horizontal axis shows the length of mature miRNA, and the vertical axis represents for percentage.
